# UHPLC-MS/MS-Based Identity Confirmation of Amino Acids Involved in Response to and Side Effects from Antiseizure Medications

**DOI:** 10.1021/acs.jproteome.2c00835

**Published:** 2023-02-22

**Authors:** Mo Awchi, Pablo Sinues, Alexandre N. Datta, Diego García-Gómez, Kapil Dev Singh

**Affiliations:** † University Children’s Hospital Basel, University of Basel, Spitalstrasse 33, 4056 Basel, Switzerland; ‡ Department of Biomedical Engineering, University of Basel, Gewerbestrasse 14, 4123 Allschwil, Switzerland; § Department of Analytical Chemistry, University of Salamanca, Plaza de los Caídos s/n, 37008 Salamanca, Spain

**Keywords:** UHPLC, tandem mass spectrometry, compound identification, exhaled breath condensate, amino acids

## Abstract

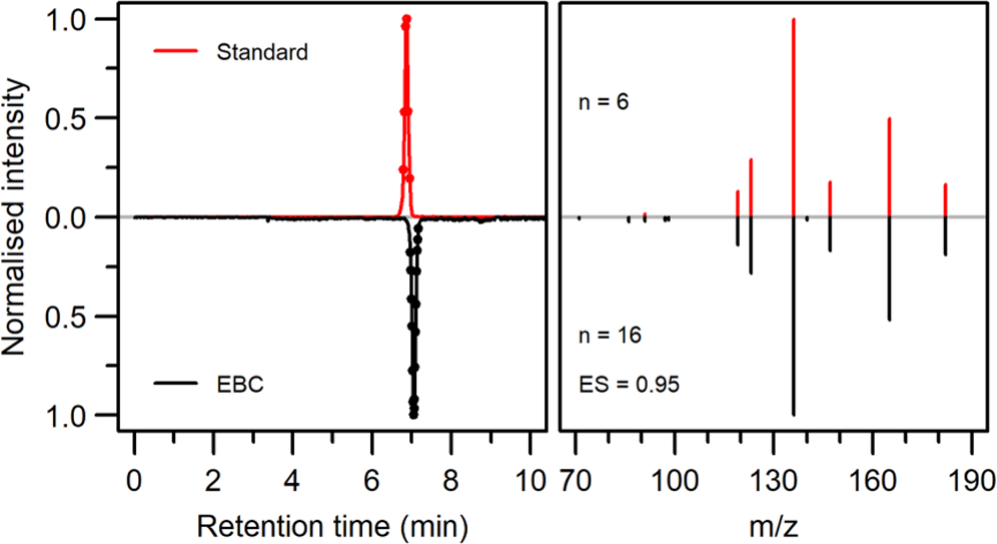

Real-time breath analysis using secondary electrospray ionization coupled with high-resolution mass spectrometry is a fast and noninvasive method to access the metabolic state of a person. However, it lacks the ability to unequivocally assign mass spectral features to compounds due to the absence of chromatographic separation. This can be overcomed by using exhaled breath condensate and conventional liquid chromatography–mass spectrometry (LC–MS) systems. In this study, to the best of our knowledge, we confirm for the first time the presence of six amino acids (GABA, Oxo-Pro, Asp, Gln, Glu, and Tyr) previously reported to be involved in response to and side effects from antiseizure medications in exhaled breath condensate and by extension in exhaled human breath. Raw data are publicly available at MetaboLights with the accession number MTBLS6760.

## Introduction

During the last decade, real-time exhaled breath analysis developed as a promising tool for personalized medicine. In addition to respiratory gases and water vapor, exhaled breath contains a minuscule amount of various volatile organic compounds (VOCs), of both endogenous and exogenous origins.^1^ The composition and concentration of these VOCs give rise to the characteristic “breath-print” of an individual, which provides relevant metabolic information and makes exhaled breath analysis an attractive tool for clinical diagnosis. Moreover, in addition to being noninvasive and quick, the use of real-time analysis methods eliminates sample handling and processing inconsistencies. All of these advantages allow monitoring and quantification of various fatty acids,^2^ amino acids,^3^ and TCA metabolites^4^ among others in the past using real-time breath analysis. Additionally, real-time breath analysis can be employed to perform drug pharmacokinetics^5^ and phenotype lung diseases such as asthma^6^ and chronic obstructive pulmonary disease.^7,8^ Recently, we reported the successful implementation of real-time breath analysis using secondary electrospray ionization coupled with high-resolution mass spectrometry (SESI-HRMS) to provide reliable estimations of systemic drug concentrations along with risk estimates for drug response and side effects in epileptic patients,^9^ where risk estimates were based on features with differential abundance between either (i) patients suffering from side effects against no side effects or (ii) patients not responding to their antiseizure medications (ASMs) against the responders. Additionally, features were assigned to compounds based on accurate mass, revealing the upregulation of serval amino acids (such as GABA, Asp, Gln, Glu, Pro, Lys, etc.) in patients suffering from side effects and downregulation of tyrosine metabolic pathway compounds (such as Tyr, Phe, dopamine, etc.) in nonresponders.^9^

Despite the promising potential, one of the main disadvantages of real-time breath analysis is its inability to unequivocally confirm mass spectral features to compound assignment, which is crucial to make it transition into clinics.^10^ However, this can be overcomed by using exhaled breath condensate (EBC)^11^ and conventional LC–MS/MS to achieve “Level 1” compound identifications.^12^ Here, we used EBC and LC–MS/MS to confirm six previously mentioned compound assignments based on accurate mass from our recent work.^9^

## Experimental Section

### Chemicals

Pure chemical standards of selected amino acids, i.e., γ-aminobutyric acid (GABA), 5-oxo-proline (Oxo-Pro), aspartic acid (Asp), glutamine (Gln), glutamic acid (Glu), and tyrosine (Tyr), were purchased from Sigma. All other LC solvents such as water, acetonitrile (ACN), and methanol with and without 0.1% formic acid (FA) were also purchased from Sigma. All solvents used were of at least LC–MS grade.

### Exhaled Breath Condensate Sampling and Upconcentration

EBC was collected using a custom-built sampling device (containing dry ice and isopropanol^13^) based on the guidelines of the ATS/ERS task force.^11^ In total, 100 mL of EBC was collected after pooling samples from six healthy (three males and three females) subjects. On average, to collect 10 mL of EBC, a subject had to exhale for 1.5 h. All subjects signed an informed consent prior to sampling, and the study was approved by the local ethics committee for Northwest and Central Switzerland (2018-01324).

Collected EBC was then upconcentrated using a vendor-recommended solid-phase extraction (SPE) procedure. Briefly, the Oasis hydrophilic–lipophilic balance (HLB) cartridge (6 cc, 150 mg, 30 μm) from Waters (Milford) was first preconditioned using 2 mL of methanol and 2 mL of water. Thereafter, 100 mL of sample was loaded on the cartridge and subsequently eluted using 2 mL of methanol without any intermediate washing. All solvents and sample were allowed to flow through the cartridge under gravity. The methanol from the eluent was evaporated under a gentle stream of nitrogen before reconstituting it in 500 μL of water (i.e., ∼200 times theoretical upconcentration).

### Compound Selection and Standard Solution

Extracted ion chromatograms (XICs) of features corresponding to compounds involved in response to and side effects from ASMs,^9^ from the EBC sample, were screened to select the initial set of compounds (Figure S1). Afterward, only compounds with commercially available pure chemical standards were kept (Figure S1). All standards were first dissolved in water to create individual master stock solutions of concentration 0.1 mg/mL. Finally, all standard stock solutions were serially diluted 10 000 times (except for Asp, which was diluted to only 100 times) to achieve the final signal between 10^6^ and 10^8^ orders of magnitude at the injection volume of 100 μL.

### UHPLC-MS/MS

Data-dependent tandem MS (MS/MS) experiments were performed on a Q Exactive Plus mass spectrometer with a heated electrospray ionization (HESI) source (Thermo Fisher Scientific, Germany) coupled with an ultrahigh-performance liquid chromatography (UHPLC) system (Vanquish, Thermo Fisher Scientific, Germany). Then, 100 μL of samples (either diluted standard solution or upconcentrated EBC) was separated on a reverse-phase analytical column with embedded weak acidic ion-pairing groups (Primesep 200 from SIELC Technologies, 150 × 4.6 mm^2^ ID, 5 μm) at a flow rate of 0.7 mL/min maintained at 25 °C. Samples were eluted with a gradient between solvent A (water with 0.1% FA) and solvent B (ACN with 0.1% FA). The gradient profile was 15% solvent B between 0 and 2 min, 15–95% solvent B between 2 and 15 min, 95% solvent B between 15 and 20 min, followed by column re-equilibration to 15% solvent B in a total of 24 min run (including 2 min of preinjection equilibration).

Eluted samples were directly ionized in the HESI source (see Table S1 for tune settings). The mass spectrometer was operated in the positive polarity full MS/dd-MS^2^ (top 5) mode with one full scan in Orbitrap (scan range = 70–400 *m*/*z;* resolution = 140 000 (at 200 *m*/*z*); AGC target = 10^6^; max. injection time = 200 ms), followed by higher-energy collisional dissociation (HCD) fragmentation of the five most intense ions (intensity threshold = 10^4^; isolation window = 1 *m*/*z*; NCE = 30; dynamic exclusion = auto), and acquisition of MS/MS spectra in Orbitrap (resolution = 70 000 (at 200 *m*/*z*); AGC target = 10^6^; max. injection time = 100 ms). Additionally, as previously recommended,^14^ a static exclusion list consisting of features with average intensity higher than 10^4^ from three blank runs was also used. The mass spectrometer was externally calibrated prior to the measurement using a commercially available calibration solution (product number 88340, Pierce Triple Quadrupole, extended mass range, Thermo Fisher Scientific, Germany) and internally calibrated using *m*/*z* 279.15909 (dibutyl phthalate, a common plasticizer^15,16^) as lock mass during measurements.

The raw data files are publicly available at the MetaboLights (https://www.ebi.ac.uk/metabolights) repository,^17^ with the accession number MTBLS6760.

### Data Analysis

The data for XICs and MS/MS spectra plotted in various figures were directly retrieved from the RAW files using an in-house C# console app based on RawFileReader (version 5.0.0.38, an open-source. Net assembly) from Thermo Fisher Scientific. Afterward, all figures were plotted using R (version 4.1.0).^16^

## Results and Discussion

### Detection of Amino Acids in Exhaled Breath by SESI-HRMS

**Figure 1:**
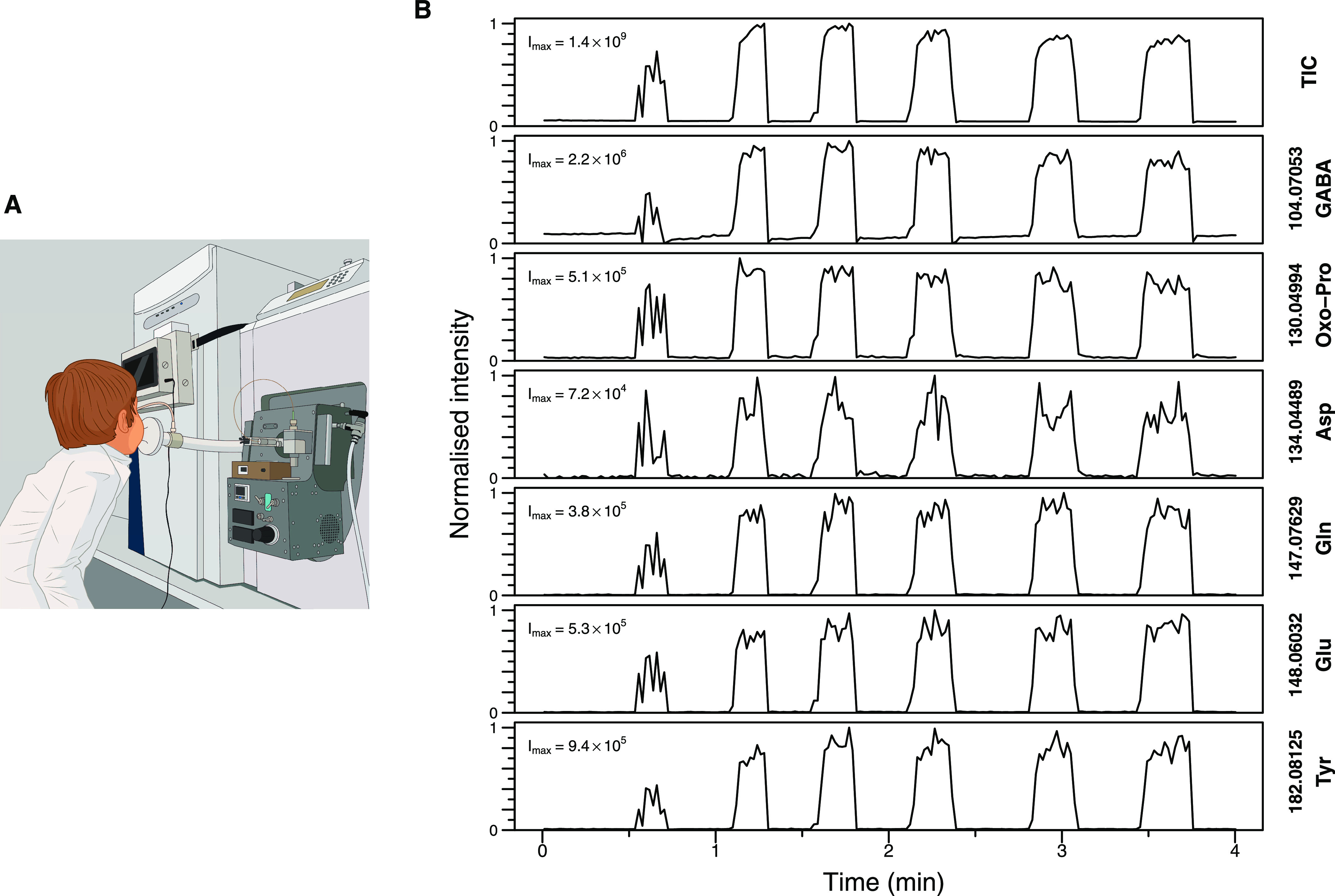
Detection of selected amino acids in exhaled breath. (A) Pictorial representation of a patient exhaling into the SESI-HRMS analytical setup. (B) Total ion current and traces of features assigned to the protonated adduct of six selected amino acids as measured in real-time exhaled breath.

Recently, we showed that in epileptic patients, real-time breath analysis could be used to provide risk estimates for response to and side effects from antiseizure medication (ASM).^9^ However, these estimates were based on mass-to-charge (*m*/*z*) ratios (i.e., features) assigned to compounds from various amino acid metabolic pathways based solely on measured accurate mass, which prompted us to caution readers until unambiguous chemical identification is provided (for more details, see Figure 6a and Supplementary Data 6 from ref (9)). Here, we selected six amino acids (Table 1) previously reported to be involved in response to and side effects from ASMs, for further compound confirmation based on UHPLC-MS/MS. Figure 1A shows a typical real-time SESI-HRMS measurement setup, whereby a subject exhales 5–6 times directly into a SESI source coupled with HRMS. The whole procedure usually lasts 5 min per polarity mode. Figure 1B shows the real-time traces for features assigned to the protonated adduct of the six compounds of interest from one measurement of a previous study as an example.

**Table 1:** Selected Amino Acids with Assigned Features

Compound		Identification		
ID	Name	*m*/*z* (positive polarity)	Adduct^a^	Confidence code^b^
C00334	γ-Aminobutyric acid (GABA)	104.07053	B	1
C01879	5-Oxoproline (Oxo-Pro)	147.07629 | 130.04994 | 113.02330 | 112.03930	A | B | C | D	NA | 1 | NA | NA
C00049	l-Aspartic acid (Asp)	134.04489 | 116.03422	B | D	1 | 2d
C00064	l-Glutamine (Gln)	147.07629 | 130.04994 | 129.06587	B | C | D	1 | 2d | NA
C00025	l-Glutamic acid (Glu)	148.06032 | 131.03395 | 130.04994	B | C | D	1 | NA | 2d
C00082	l-Tyrosine (Tyr)	182.08125 | 165.05467 | 164.07056	B | C | D	1 | 2d | NA

a In adduct: A = [M + NH_4_]^+^; B = [M + H]^+^; C = [M – NH_3_ + H]^+^; and D = [M – H_2_O + H]^+^.

b In confidence code: 1 = level 1 (RT, MS/MS, reference standard) and 2d = level 2d (RT, reference standard); NA = not assigned.

### Confirmation of Amino Acid Presence in EBC

**Figure 2:**
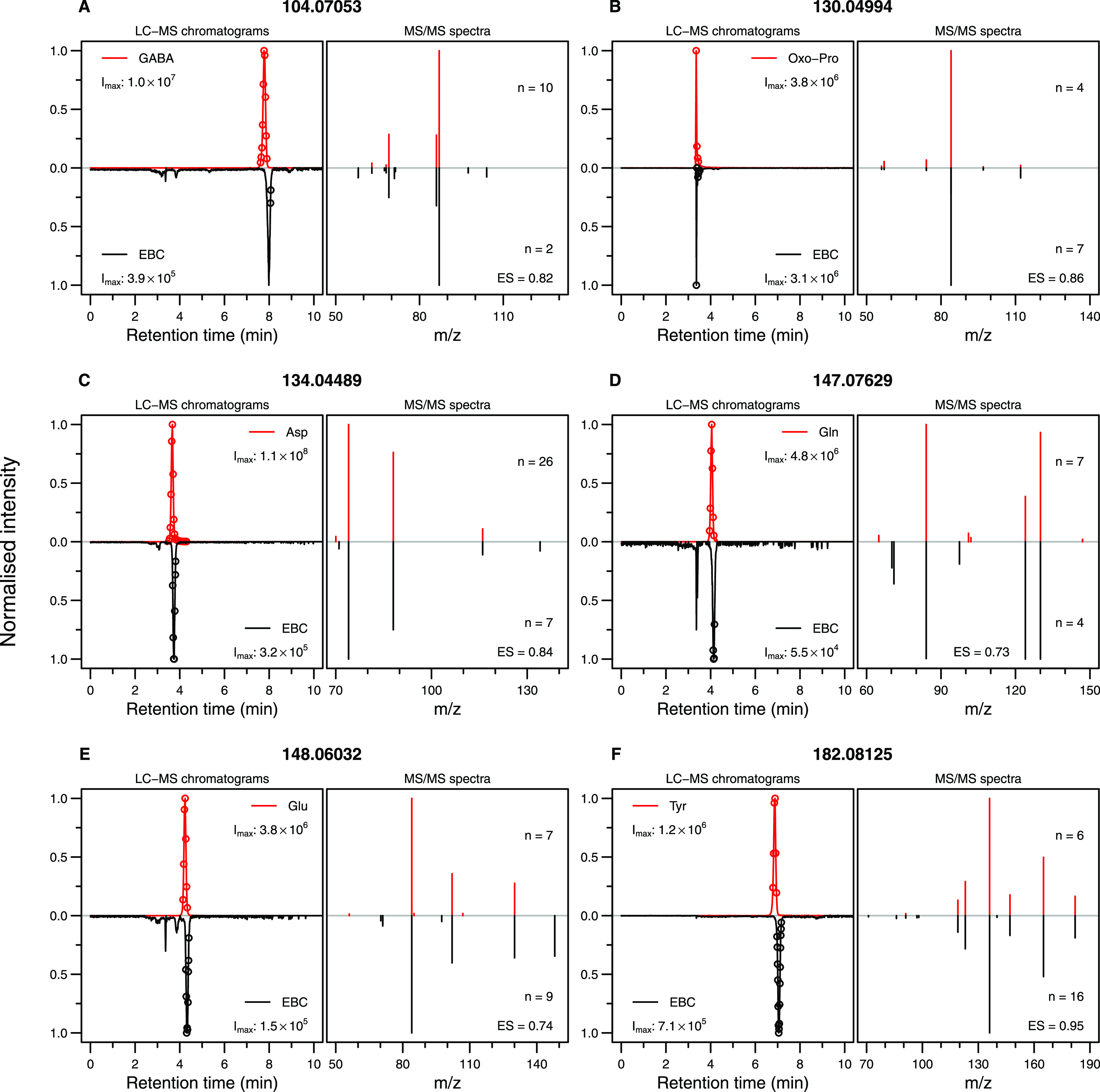
UHPLC-MS/MS confirmation of protonated adducts of selected amino acids. The figure shows the compound identification via UHPLC-MS; we confirmed that *m*/*z* 104.07053 belongs to protonated GABA (A), *m*/*z* 130.04994 belongs to protonated Oxo-Pro (B), *m*/*z* 134.04489 belongs to protonated Asp (C), *m*/*z* 147.07629 belongs to protonated Gln (D), *m*/*z* 148.06032 belongs to protonated Glu (E), and *m*/*z* 182.08125 belongs to protonated Tyr (F). Each panel shows the compression of LC–MS chromatograms (i.e., XICs) and the average MS/MS spectra between the pure standards of suspected molecules and EBC. The number of MS/MS spectra used to obtain the averaged MS/MS spectrum is denoted by *n*, and the time at which those MS/MS spectra were triggered is denoted by the open circles in the corresponding LC–MS chromatograms. The MS/MS spectrum similarity score based on spectral entropy similarity between standards and EBC is denoted by ES.

Different features were previously assigned to six selected amino acids owing to different adduct forms (Table 1). Initially, we focused on the protonated adduct form, whereby matching the retention time (RT) and MS/MS spectra between pure chemical standards and EBC in a UHPLC-MS/MS experiment unequivocally confirmed previous features to compound assignment (Figure 2). The spectrum similarity score, between the MS/MS spectra originating from standards and EBC, was calculated for all compounds based on the spectral entropy similarity (ES) method.^18^ Additionally, we compared the retention time of other adduct forms between standards and EBC for all six compounds (Figure S2). For some adduct forms, due to low signal intensity, no clear peak was observed either in standards (Figure S2A,I) or in EBC (Figure S2K) or sometimes in both (Figure S2C,D,O), suggesting the possibility that these ions are unstable in conventional HESI settings as compared to the real-time environment of the SESI source. Table S1 shows the comparison of tune settings between the HESI source used during UHPLC-MSMS analysis of this study and the SESI source used during real-time breath analysis from the previous study.^9^ Although most of the source-specific tune settings have little to no impact on the ion stability, recently, it was shown that higher capillary temperature leads to “hard” ionization.^19^ Furthermore, whenever a clear peak was observed for different adduct forms in the standard of the same compound, it had the same retention time, indicating that adduct formation happens after chromatography, perhaps in-source. In addition to the aforementioned confirmation of protonated forms, Figure S2F confirms *m*/*z* 116.03422 as dehydrated Asp, whereas Figure S2N confirms *m*/*z* 165.05467 as loss of ammonia adduct of Tyr.

From Table 1, it is clear that *m*/*z* 130.04994 and 147.07629 were assigned to more than one compound based on different adduct forms. Furthermore, XIC for *m*/*z* 147.07629 in EBC has two resolved peaks (Figures 2D and S3, same EBC trace), where the peak on the right has been established to correspond to protonated Gln (Figure 2D). Unfortunately, as mentioned above, no clear peak was observed in the standard for the ammonium adduct of Oxo-Pro (Figures S2A and S3A), but interestingly, the left peak has the same retention time as the standard of protonated Oxo-Pro. Hence, based on the fact that theoretically, the ammonium adduct of Oxo-Pro will also have the same retention time as the protonated form of Oxo-Pro, we deduced that the left peak in EBC probably corresponds to the ammonium adduct of Oxo-Pro (Figure S3A). Similarly, in the measured EBC sample, *m*/*z* 130.04994 mainly corresponds to protonated Oxo-Pro. However, a small fraction of this feature may also reflect dehydrated Glu and/or loss of the ammonia adduct of Gln (Figure S3B).

This prompted us to look back into the real-time breath signal of these two features from the previous epileptic data set,^9^ and as now expected, we observed two distinct clusters in the breath levels of *m*/*z* 147.07629 (Figure S3A inset in the EBC trace). Furthermore, matching with the UHPLC data, breath levels for *m*/*z* 130.04994 show indistinct clusters (Figure S3B inset in the EBC trace). These observations re-emphasize that by itself real-time SESI-HRMS is unable to resolve in-source fragments and adducts of different compounds that leads to the same feature; hence, one must remain cautious while working with uncharacterized features. Furthermore, this also necessitates the use of chromatographic separation for accurate feature characterization.^20^

## Conclusions

As mentioned in Table 1, we concluded that for the selected compounds all of the assignments with protonated adduct form can be confirmed at “Level 1” confidence (i.e., matching RT and MS/MS spectra with reference standards). Whereas, for assignments with other adduct forms , we could either reach only “Level 2d” confidence “NA” (i.e., only matching RT with reference standards) or could not assign any confidence code (i.e., NA).^12^ It must be noted that confidence code does not mean a wrong assignment; it merely reflects the fact that we were unable to confirm the assignment. Additionally, one must not forget that during a real-time SESI measurement, a single mass spectral feature could represent two or more adduct forms of different compounds, as observed here in the case of *m*/*z* 147.07629 and 130.04994. Finally, simple confirmatory studies like this are essential to develop and maintain comprehensive breath features to the compound database, which will ultimately help breath research transition from the bench to the market.

## Supporting Information Available

Table S1, comparison of tune settings between the HESI source used during UHPLC-MSMS analysis of this study and the SESI source used during real-time breath analysis from the previous study; Figure S1, workflow used to select compounds for the UHPLC-MSMS-based confirmation; Figure S2, comparison of LC–MS chromatograms between standards of six selected compounds and EBC for different adduct forms, as defined in Table 1; and Figure S3, features at *m*/*z* 147.07629 and 130.04994 appear to be associated with different adduct forms of more than one compound (PDF)

## Notes

P.S. is a cofounder of Deep Breath Intelligence AG (Switzerland), which develops breath-based diagnostic tools. K.D.S. is a consultant for Deep Breath Intelligence AG (Switzerland).
