# Supplementary material for: UHPLC-MS/MS-Based Identity Confirmation of Amino Acids Involved in Response to and Side Effects from Antiseizure Medications
Source: J Proteome Res. 2023 Feb 22;22(3):990–5. doi: 10.1021/acs.jproteome.2c00835 (PMC9990125; doi:10.1021/acs.jproteome.2c00835)
Supplement: Supplementary file 1 — pr2c00835_si_001.pdf [file pr2c00835_si_001.pdf]

UHPLC-MS/MS based identity confirmation of amino acids involved in response to and side effects from antiseizure medications

Mo Awchi[1,2], Pablo Sinues[1,2], Alexandre N. Datta[1], Diego García-Gómez[3], Kapil Dev Singh[1,2,*]

1. University Children's Hospital Basel, University of Basel, Basel, Switzerland.

2. Department of Biomedical Engineering, University of Basel, Basel, Switzerland.

3. Department of Analytical Chemistry, University of Salamanca, Salamanca, Spain.

* Corresponding author: kapil.singh@ukbb.ch

Table of Contents

Table S1. Comparison of tune settings between the HESI source used during UHPLC-MSMS analysis of this study and the SESI source used during real-time breath analysis from the previous study. S-3

Figure S1. Workflow used to select compounds for UHPLC-MSMS based confirmation. S-4

Figure S2. Comparison of LC-MS chromatograms between standards of six selected compounds and EBC for different adduct forms, as defined in Table 1. ....S-5

Figure S3. Features at m/z 147.07629 and 130.04994 appear to be associated with different adduct forms of more than one compound. S-6

	Settings	HESI source	SESI source
Source specific	Sheath gas flow rate	10	60
	Aux gas flow rate	0	2
	Sweep gas flow rate	0	0
	Spray voltage (|kV|)	4	3.5
	Probe heater temp. (°C)	30	-
	Sampling line temp. (°C)	-	130
	Core temp. (°C)	-	90
MS specific	Capillary temp. (°C)	320	275
	S-lens RF level	55	55

Table S1. Comparison of tune settings between the HESI source used during UHPLC-MSMS analysis of this study and the SESI source used during real-time breath analysis from the previous study.

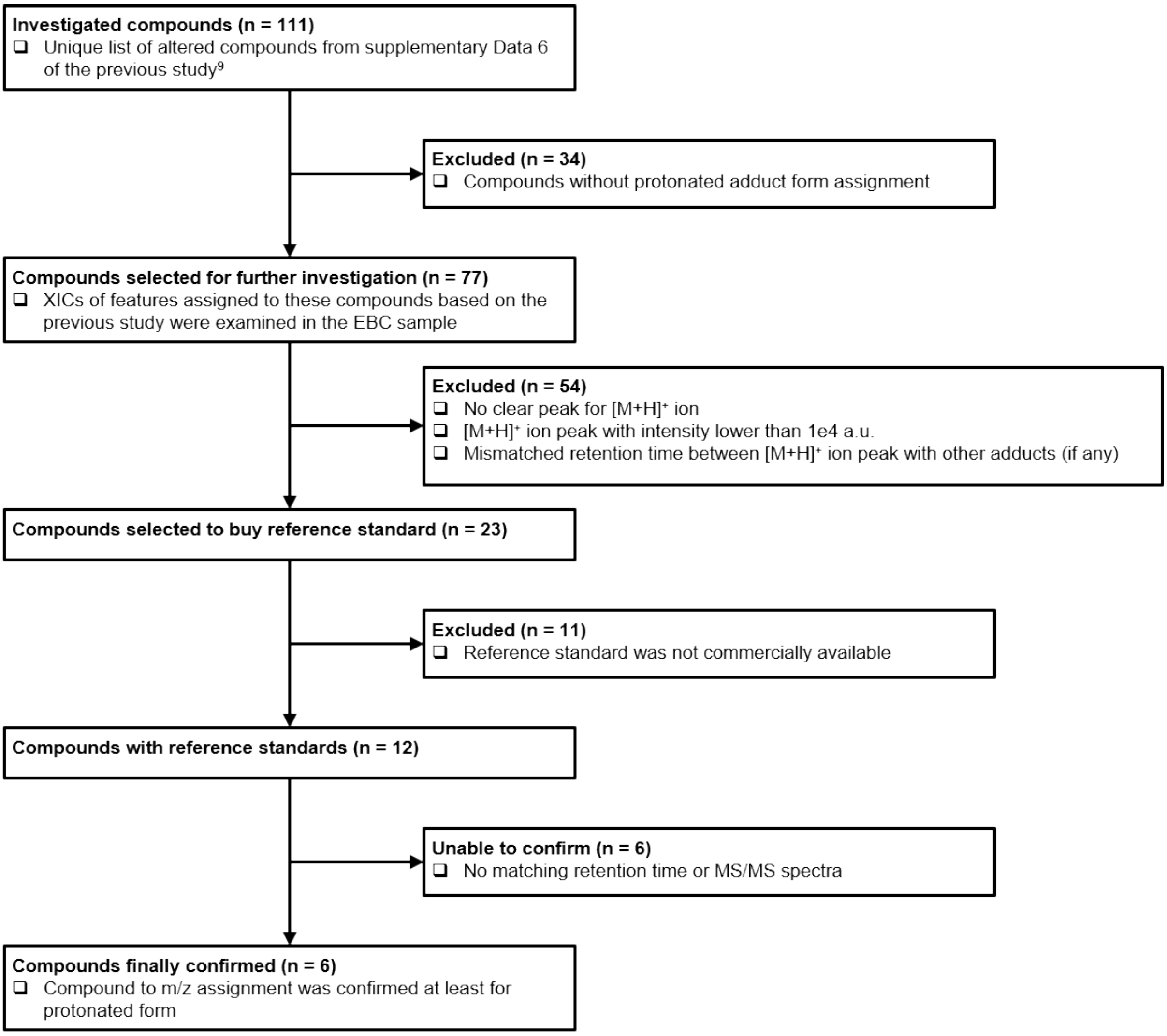

Figure S1. Workflow used to select compounds for UHPLC-MSMS based confirmation.

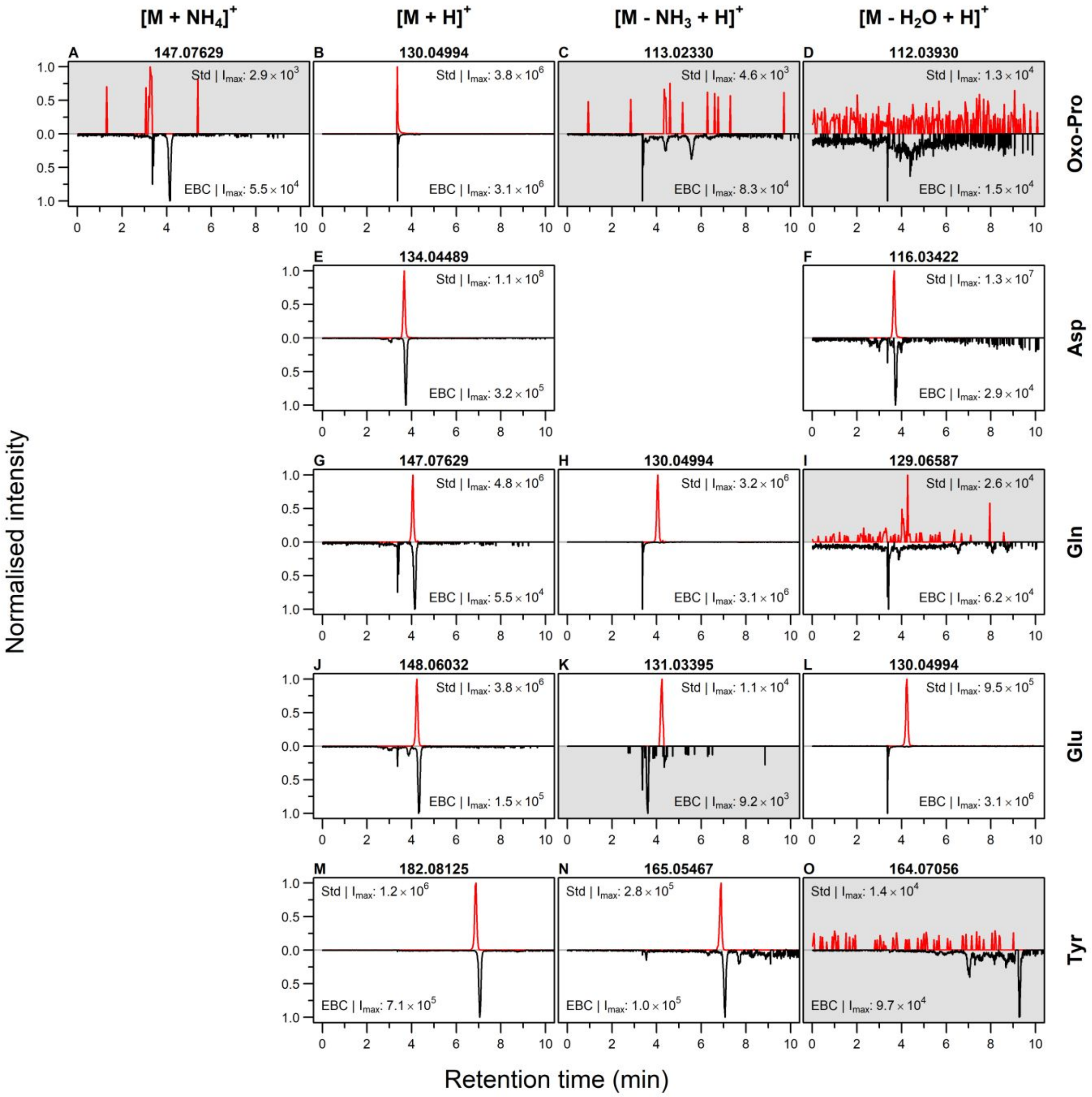

Figure S2. Comparison of LC-MS chromatograms between standards of six selected compounds and EBC for different adduct forms, as defined in Table 1. Grey background points to the LC-MS chromatogram (i.e. XIC) with no clear peak.

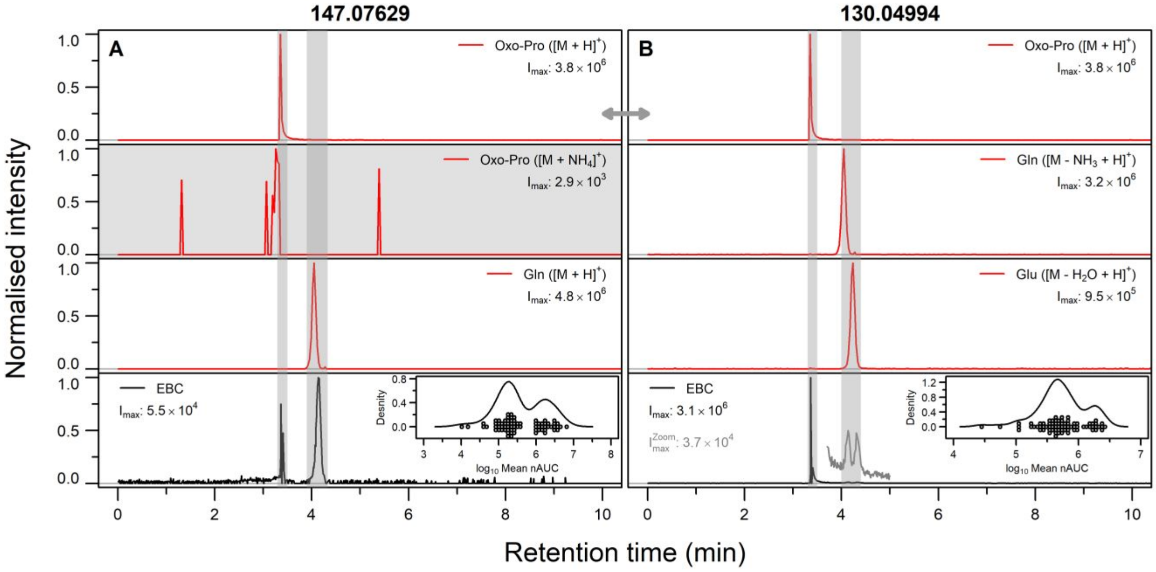

Figure S3. Features at m/z 147.07629 and 130.04994 appear to be associated with different adduct forms of more than one compound. The figure shows the comparison of LC-MS chromatograms between different standards (with different adduct forms) and EBC. Chromatograms shown here are same as the corresponding ones from Figure S2 (just rearranged here to ease comparison). Like Figure S2, chromatograms with no clear peak are shown with grey background. Furthermore, peaks in the XIC of EBC are highlighted with grey vertical bars to ease comparison. Finally, the grey trace in the EBC of panel B around 4 min represents ~100 times zoomed XIC in the y-axis, to reveal two smaller peaks. Inset in EBC panel shows the distribution of mean normalised area under the curve (nAUC) of selected feature from previous real-time breath analysis,[9] as density curves accompanied by actual data points, suggesting that m/z147.07629 possibility corresponds to two different compounds in the previous study. Note: The chromatogram for protonated Oxo-Pro in both panel is the same XIC for m/z 130.04994 as denoted by the bidirectional arrow, see text for more details.
